# Ambient Temperature Influences Australian Native Stingless Bee (*Trigona carbonaria*) Preference for Warm Nectar

**DOI:** 10.1371/journal.pone.0012000

**Published:** 2010-08-09

**Authors:** Melanie Norgate, Skye Boyd-Gerny, Vera Simonov, Marcello G. P. Rosa, Tim A. Heard, Adrian G. Dyer

**Affiliations:** 1 School of Biological Sciences, Monash University, Clayton, Victoria, Australia; 2 Department of Physiology, Monash University, Clayton, Victoria, Australia; 3 CSIRO Entomology, Indooroopilly, Australia; Freie Universitaet Berlin, Germany

## Abstract

The interaction between flowers and insect pollinators is an important aspect of the reproductive mechanisms of many plant species. Several laboratory and field studies indicate that raising flower temperature above ambient can be an advantage in attracting pollinators. Here we demonstrate that this preference for warmer flowers is, in fact, context-dependent. Using an Australian native bee as a model, we demonstrate for the first time a significant shift in behaviour when the ambient temperature reaches 34°C, at which point bees prefer ambient temperature nectar over warmer nectar. We then use thermal imaging techniques to show warmer nectar maintains the flight temperature of bees during the period of rest on flowers at lower ambient temperatures but the behavioural switch is associated with the body temperature rising above that maintained during flight. These findings suggest that flower-pollinator interactions are dependent upon ambient temperature and may therefore alter in different thermal environments.

## Introduction

Many flowering plants predominantly rely on insect pollinators for reproduction [1]. To maximise the efficiency of pollen dispersal plants may offer small nutritional rewards that encourage insects to make successive visits to a particular type of flower, a phenomenon termed flower constancy [2]. Raising intrafloral temperature, often beneficial for flower development [3], also appears to encourage a wide variety of insects to visit and spend time in flowers [4]–[9]. Warmth from flowers can provide an energetic benefit to visiting pollinators [4], [5], [10], and modelling suggests this may allow plants to provide the same energetic reward with less nectar production [11]. Several examples of a link between floral temperature and nectar quantity and quality [7], [12] suggest this combination is a possible driver for flower constancy.

Bees are one of the most prolific pollinators of flowering plants because they are extremely adept at learning different flower features [13], [14]. Nutrition coupled with warmth could entice bees to preferentially visit certain flowers if they were able to perceive the additional temperature reward in the context of nutritional rewards. This reward model has been demonstrated for bumblebees (*Bombus terrestris*) tested in 18.5°C laboratory conditions using artificial flowers with identical nutritional rewards but varying in temperature [15]. Importantly from an ecological point of view, individual bumblebees were able to use secondary cues like colour to preferentially visit the warmer flower type. This indicates that the reward model of warmth coupled with nutrition could significantly influence pollinator choices in complex natural environments through associative learning [15]. In addition, temperature and perceived sucrose sweetness are processed independently by bumblebees, indicating that flower temperature is a distinct reward for pollinators [16].

The suggestion that energetic benefit is a likely driver for warmth preference raises two important questions. First, bee species vary in body size and foraging temperatures, so it is important to know if species other than bumblebees (which are among the largest of bees) also prefer warmer nectar. Second, it is important to investigate how bee preferences for warmer flowers might be affected by ambient temperature, given that insect flight is energetically expensive and can be maintained only within a defined thoracic temperature range [17]. If the preference for warmer nectar is mediated by a mechanism such as taste reception, we can expect a consistent linear (or logarithmic) response to increasing sucrose temperature. However, if nectar temperature has an impact on insect thermoregulation we should observe a change in behaviour as the warmer nectar exceeds the temperature that is beneficial for the bee.

This study investigates the dynamic range of nectar temperature preference in the Australian native stingless bee, *Trigona carbonaria*. Stingless bees are typically smaller and occur in warmer climates than bumblebees [18], [19] but, like bumblebees, *T. carbonaria* are a potentially important pollinator for several major commercial crops and many wild plant species [20]. Comparing these two bee species therefore provides an indication of how widespread nectar temperature preferences are amongst Apinae. In addition, examining the interaction of pollinator temperature preferences with ambient temperature allows for an understanding of the potential for temperature variation to affect plant-pollinator interactions.

## Methods

### Laboratory environment

Experiments were conducted at Monash University between November 2008 and February 2009 in a 3×5 m controlled temperature laboratory, allowing the temperature to be set between 4 and 40°C with relative humidity set to 30%. Between experiments bees were maintained at a constant temperature of 23°C. Illumination (10/14 h day/night) was provided by four Phillips Master TLS HE slimline 28 W/865 UV+ daylight fluorescent tubes (Holland) with specially fitted high frequency (>1200 Hz) ATEC Jupiter EGF PMD2×14–35 electronic dimmable ballasts. The flight arena (1.2×0.6×0.5 m; LWH) was made of a coated steel frame with laminated white wooden side panels. The arena floor was painted foliage green, and the arena lid was UV transparent plexiglass. This set-up approximately matches natural illumination foraging conditions for insect pollinators [21].

A research colony (*ca*. 4000 adults and 800 foraging individuals) of *T. carbonaria* was propagated [22] and established in a 27.5×20×31 cm (LWH) pine nesting box which was connected to the foraging arena by a 16 cm plexiglass tube.

### Experiment 1: Evaluation of relative bee numbers active in a laboratory environment

To ascertain that the activity level of *T. carbonaria* in the laboratory environment was approximately equivalent to that in natural conditions [23], the number of individual bees leaving the nesting box and entering the flight arena was counted. This method was identical to that used to measure activity levels in a field setting [23]. For each ambient temperature, data were collected over 20 replicates of 5 minutes taken at random intervals between the times of 1100 hours and 1400 hours, which is the time of peak bee activity in the field when measured using the same approach [23].

### Experiment 2: The dynamic range of pollinator preference for warmer flowers

The choice frequency of bees for either warm or cool feeders that presented 15% (vol.) sucrose solution was tested at four ambient temperatures (23°C, 28°C, 30°C and 34°C), which lies in the range of temperatures at which stingless bees forage [23], [24]. At this concentration studies on nine bee species show that viscosity changes do not affect bee ingestion rates [25], and that variations in sucrose temperature fall into a range where ingestion rates are independent of viscosity [15]. In each experimental condition the ‘cool’ feeder was always at the ambient temperature, whilst the warm feeder was approximately 6°C warmer (Table 1). The bees were tested first at 23°C to establish that the previously observed preference in bumblebees for a feeder that presents warmer nectar [15] also existed for *Trigona* bees. The other three temperatures were tested in a random order to control for temporal effects, with the bees allowed a minimum of 24 h habituation to a temperature condition prior to a test.

**Table 1:** Temperatures used in this study (± s.d.).

Ambient temperature	Ambient feeder temperature	Warm feeder temperature
23°C	22.9**±0.3**	29.4**±1.0**
28°C	28.1**±0.5**	34.9**±0.6**
30°C	30.3**±0.5**	36.1**±0.5**
34°C	33.6**±1.1**	38.4**±1.0**

Two identical temperature blocks (DB3-221-D 250 W; Thermoline Scientific Equipment Pty Ltd, Smithfield, NSW, Australia) were used to control the temperature of the feeders. Two visually identical gravity feeders were placed on the blocks, and the area around the gravity feeder was insulated with 10 mm foam so that only the sucrose inside the feeder was heated [15], [16]. Ambient and surface temperature was measured with a Digitech QM-1600 type-K thermocouple probe [15], [16]. The position of the warm and cool feeder was varied in a pseudo-random fashion (avoiding three consecutive tests in the same position) during experiments to control for potential side preferences. Prior to and between tests the feeders and the arena were cleaned with 10% ethanol to remove any olfactory cues.

Bees were allowed a minimum of 2 h to collect sucrose from the feeders before data collection. The dependent variable was choice frequency for the warm versus cool feeder over 1 h considering the independent variable of ambient temperature. A total of 12 replicates were completed for each of the four test conditions. The data were analysed following an arc-sine square root transformation [26], which yielded a normal distribution for every temperature and equal variance for 23°C, 28°C and 30°C (Levene's test, P>0.05).

To exclude the possibility of preferences for one side of the room that may influence the results, this experiment was also conducted using two identical ambient temperature feeders at 28°C.

**Figure 1:**
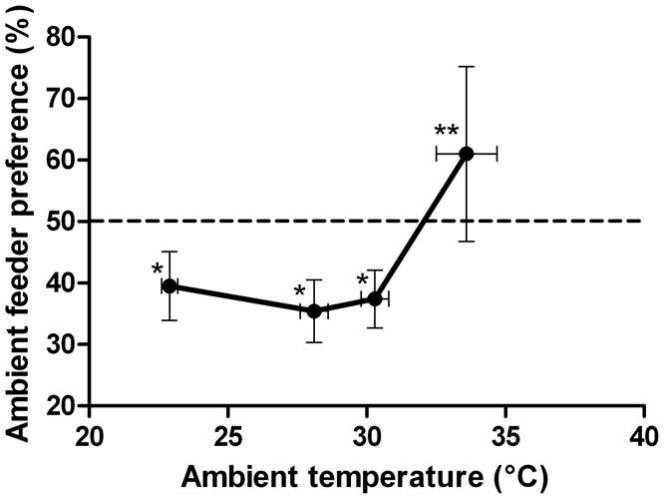
Sucrose temperature preferences of *Trigona carbonaria* across a range of ambient temperatures. The preference for a feeder that is at ambient temperature compared with a feeder that is 6°C warmer is dependent upon the ambient temperature (mean +/− s.d.). *Preference for the ambient feeder is significantly lower than expected by chance (one sample t-test, P<0.001), but the preferences do not differ significantly different from each other [one way ANOVA, F(2,33)  = 1.527, P = 0.232]. **Preference for the ambient feeder is significantly higher than expected from chance (one sample t-test, t = 2.635, DF = 11, P = 0.023).

Statistical analyses were conducted using SPSS v15. For each ambient temperature, a one sample t-test was performed to compare the observed preference to that expected by chance. For ambient temperatures 23°C, 28°C and 30°C, at which the preferences were all for the warmer feeder, a one-way ANOVA was then performed to investigate whether or not they differed from each other. The results of these analyses are shown in the Fig. 1 legend.

### Experiment 3: Distinction between temperature preference and aversive stimulus avoidance

It is possible that bees land to feed but avoid imbibing the undesirable sucrose once they detect the temperature. To investigate whether or not the sucrose had been imbibed, green food dye (Queen Foods no. 090086, Qld, Australia) and red dye (no. 725085) were used to colour the cool and warm feeder sucrose, respectively, at an ambient temperature of 33°C, where the warmer feeder was 38°C. Whilst red and green colours are easily discriminated by trichromatic human vision due to the close spectral separation of our medium (534 nm) and long wavelength (564 nm) receptors [27], these stimuli are similar for bee colour vision as only the long wavelength receptor is strongly sensitive to red/green wavelengths [28]. Therefore bees are unlikely to discriminate these colours unless specifically trained to do so with differential conditioning [29]. Individual bees (n = 18) that had visited the respective feeders were anaesthetised with CO_2_ to induce regurgitation of the coloured crop (honey stomach) contents onto filter paper. The relative size of a regurgitated droplet was compared to 1–5 µl drops from a micropipette by recording droplet surface areas with a digital camera, and using Adobe Photoshop CS2 version 9.0 software to quantify and compare the size of the respective droplets.

### Experiment 4: Thermal imaging of bees imbibing warm nectar

To further investigate the potential benefits or disadvantages of a bee of imbibing warm nectar under various conditions, we used a thermal camera (Model FLIR i50: FLIR Systems, Notting Hill Victoria, Australia) to image individual bees immediately after engaging in one of four activities: (i) resting for at least 5 min, (ii) flying, (iii) drinking sucrose from a feeder that is at ambient temperature, and (iv) drinking sucrose from a feeder that is above ambient temperature. Each of these four activities were evaluated at three ambient temperatures (23°C, 30°C and 34°C) using the same experimental conditions and nectar temperatures described in Experiment 2 for these ambient temperatures. Resting bees were measured by collecting bees from the flight arena and placing them in a 10 ml test tube for at least 5 min during which time their only activity was walking. Other bees were collected for imaging directly from the feeder or flight arena with forceps. No imaged bees were returned to the flight arena.

The accuracy with which the thermal camera could resolve temperature was empirically determined by imaging a black body radiator made from a 5 mm diameter sphere of Blue-Tack (Bostik Ltd, Thomastown, Victoria, Australia) which was then coloured matt black with Sharpie permanent marker (ACME, USA) [6]. The thermal camera was calibrated by FLIR Systems. The thermocouple probe was calibrated against the thermal camera and found to be consistent with a mercury thermometer at an ambient temperature of 24.5°C. The type-K probe was then used to measure temperature whilst embedded approximately 1 mm into the surface of the black body radiator. A heating block was used to raise the temperature of the black body radiator to approximately 38°C. The black body radiator was then removed from the heating block and immediately placed under the camera, and temperature was recorded at 5 s intervals using both the thermal camera and type-K thermocouple probe during the period (approximately 260 s) that the black body radiator cooled to the ambient temperature. The thermal camera images were processed using Adobe Photoshop CS2 v9.0 to enable a match of image pixel values [30] for the black body radiator to the calibration scale for each thermal image. Thermal camera temperature data were compared to type-K thermal probe temperature data with a total of 174 measurement replicates tested. The mean temperature difference between the two over the entire range examined for the black body radiator was 0.3°C+/− 0.2 and this accuracy was consistent within the range of 24–38°C (90% confidence interval was in the range 0.6 to 0.9°C). Thus thermal camera accuracy at collecting temperature data from a small black body radiator was determined to be better than 1.0°C.

The thermal camera was then used to image individual bees immediately after participating in one of the four activities considering the three ambient temperatures. In each trial, imaging continued with data capture at 5 s intervals until the bee temperature stabilised at the ambient temperature.

As a point of comparison to the time taken for a bee to cool once it ceases flight, the length of time taken to imbibe 15% (vol.) sucrose from an ambient temperature feeder was also measured at 28°C and 34°C. In each test condition 20 independent bees were measured.

## Results

### Experiment 1: Evaluation of relative bee numbers active in a laboratory environment

*Trigona carbonaria* are central place foragers that collect nutrition for the entire colony, thus providing a suitable model for investigating temperature preferences in a laboratory environment as well as a point of comparison to bumblebees [15], [16]. However as *T. carbonaria* has never before been used for this type of study, this experiment was performed as a control to confirm that activity levels in the laboratory environment were suitable for data collection and was equivalent to those in natural conditions. The latter is important for ensuring the biological relevance of Experiment 2, as a previous study has identified that activity of *Trigona* bees is dependent upon factors including light level and ambient temperature [23]. Activity, defined as the number of individual bees entering the flight arena, was investigated for each ambient temperature used in Experiment 2 and compared to that seen over 18–30°C under natural conditions [23]. Under the laboratory conditions used here, the bee activity (mean number of bees entering flight arena +/− s.d.) during 5 min evaluation periods was 14+/−5 (23°C), 194+/−38 (28°C), 148+/− 52 (30°C) and 176+/−63 (34°C). This was approximately an order of magnitude lower at 23°C. The lower activity may be due to the test bees being not exposed to the range of lower ambient temperatures that occurred in previous field studies [23], which may have influenced relative activity levels. However, for temperatures of 28°C and above activity was within the activity range reported for this species in a field setting (50–350 bees leaving a hive/5 min period [23].

### Experiment 2: The dynamic range of pollinator preference for warmer flowers

This experiment was designed to investigate the potential interaction between ambient temperature and insect nectar temperature preferences for *T. carbonaria*. Importantly, the method used to quantify group bee responses to sucrose temperatures (Methods) allows efficient data collection in controlled laboratory conditions, but has been previously shown to accurately represent the behaviour of individual bees visiting flowers that offer only small nutritional rewards [15]. It has also been shown that bees can use secondary cues like spatial location or flower colour to choose warmer flowers in complex settings [15], meaning that laboratory experiments provide a good model for understanding pollinator choices in natural conditions.

The *T. carbonaria* choice frequency for a warmer feeder was investigated over a range of ambient temperatures between 23 and 34°C (Fig. 1), with the warmer feeder (6°C higher than ambient) set to approximate the temperature difference for which bumblebees show a significant preference for a warmer feeder at an ambient temperature of 18.5°C [15]. For ambient temperatures of 23°C, 28°C and 30°C, there was significant preference for the warmer feeder (one sample t-test, P<0.001) which was consistent across the three temperatures (one-way ANOVA, P = 0.232). This indicates that *T. carbonaria* display a similar ∼60% preference for warmer nectar to that seen in bumblebees previously tested at low ambient temperatures [15]. However, at an ambient temperature of 34°C, *T. carbonaria* showed significant preference for the ambient-temperature feeder (one sample t-test, P = 0.023). This indicates that there is a change in the feeder temperature preference of bees between 30 and 34°C. Interestingly, there is a significant preference for the 34°C feeder regardless of whether it is the warmer feeder (at ambient temperature 30°C) or the ambient temperature feeder (with warmer feeder at 38°C).

To test for preferences for one side of the room that may influence the results, bees were also tested in this fashion using two identical ambient temperature feeders at 28°C and found to have no preference; choice for the ‘Eastern’ feeder was 50.3%+/− 2.0 which was not significantly different to chance expectation (1 sample t-test, t = 0.466, df11, p = 0.650). Thus there was no evidence of *Trigona* bees exhibiting a feeder preference if feeders did not vary in temperature; this is consistent to previous work on bumblebees in similar conditions [15].

### Experiment 3: Distinction between temperature preference and aversive stimulus avoidance

**Figure 2:**
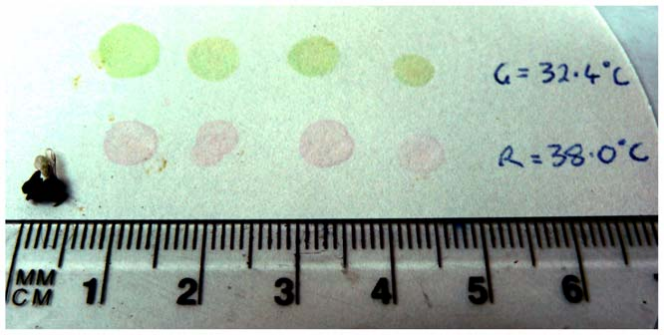
Typical crop contents of *Trigona carbonaria* bees after visiting either a cool or warm feeder. The consistency in crop contents between the two feeders indicates that the bees completely imbibe warm sucrose in the range 38–39°C. Also shown is an anaesthetised bee.

The behavioural switch observed in Experiment 2 could be mediated by an acute heat avoidance mechanism, such as when heat is used as an aversive stimulus in insect conditioning [31]. Indeed, recent work has established that honeybees can be conditioned to treat a neutral stimulus as either appetitive or aversive [32]. It is therefore important to understand if the switch in *Trigona* preferences is a response to a detrimentally hot stimulus that is perceived as aversive, in which case they might be expected to avoid imbibing the fluid once they are exposed to it. Alternatively, the preference switch could reflect flexibility in behaviour that facilitates normal thermoregulation by the bee. To distinguish between these hypotheses, the crop contents of several bees were examined immediately after they visited a feeder during the highest temperature condition (33°C ambient and 38°C warm feeder) to determine the extent to which they imbibed the fluid. Bees feeding at either a cool feeder [n = 8, mean = 2.8 µL (s.d.  = 1.2)] or a warm feeder [n = 10, mean = 2.8 µL (s.d.  = 1.0)] consistently regurgitated a similar volume of sucrose (independent samples t-test, t = 0.040, d.f.  = 16. P = 0.969; Fig. 2). Thus, once bees landed on the warm feeder, they imbibed the same volume as bees landing on the ambient temperature feeder and were not deterred by the temperature of the sucrose. This indicates that the change in behaviour at the highest ambient temperature tested is a preference switch rather than a perception of the warmest feeder as an aversive stimulus. Future experiments could help further dissect the mechanism underlying this behavioural switch.

### Experiment 4: Thermal imaging of bees imbibing warm nectar

**Figure 3:**
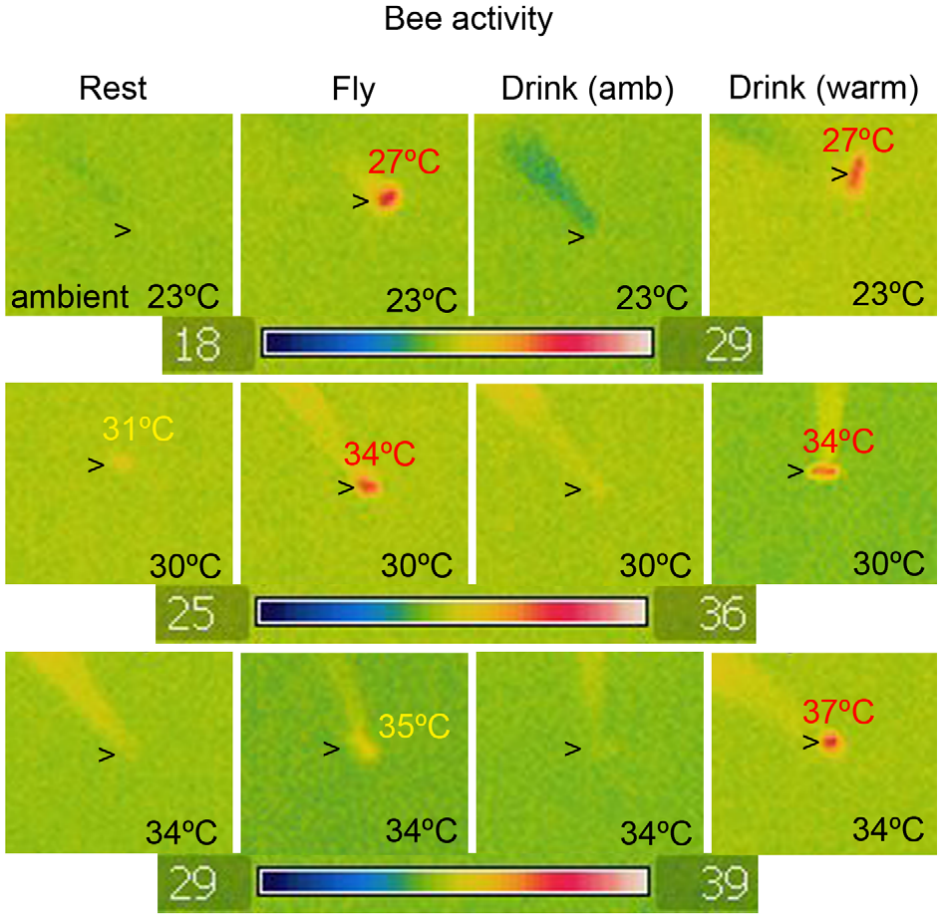
Thermal infrared images of *Trigona carbonaria* bees immediately after participating in one of four activities: Rest  =  not flying for at least 5 min, Fly  =  flying, Drink (amb)  =  imbibing sucrose from ambient feeder, and Drink (warm)  =  imbibing from warm feeder. Measurements were made with independent bees from the same colony considering three different ambient temperatures (23°C, top row; 30°C, middle row; or 34°C, bottom row). The body temperature of bees that were either resting or drinking from an ambient feeder were close to ambient temperature for all three tested ambient temperatures. However, whilst at the two lower ambient temperatures flying bees were hotter than the ambient temperature, at an ambient temperature of 34°C flying bees were close to the ambient temperature. All bees drinking from a feeder that was warmer than ambient showed a body temperature that was higher than ambient, and for an ambient temperature of 34°C this leads to a bee having a temperature that is well above the temperature bees attain during flight. Temperature scale shows temperature of bee relative to the background ambient temperature. Arrows show bee position in frame, and bee temperatures that are different from ambient are marked separately.

**Figure 4:**
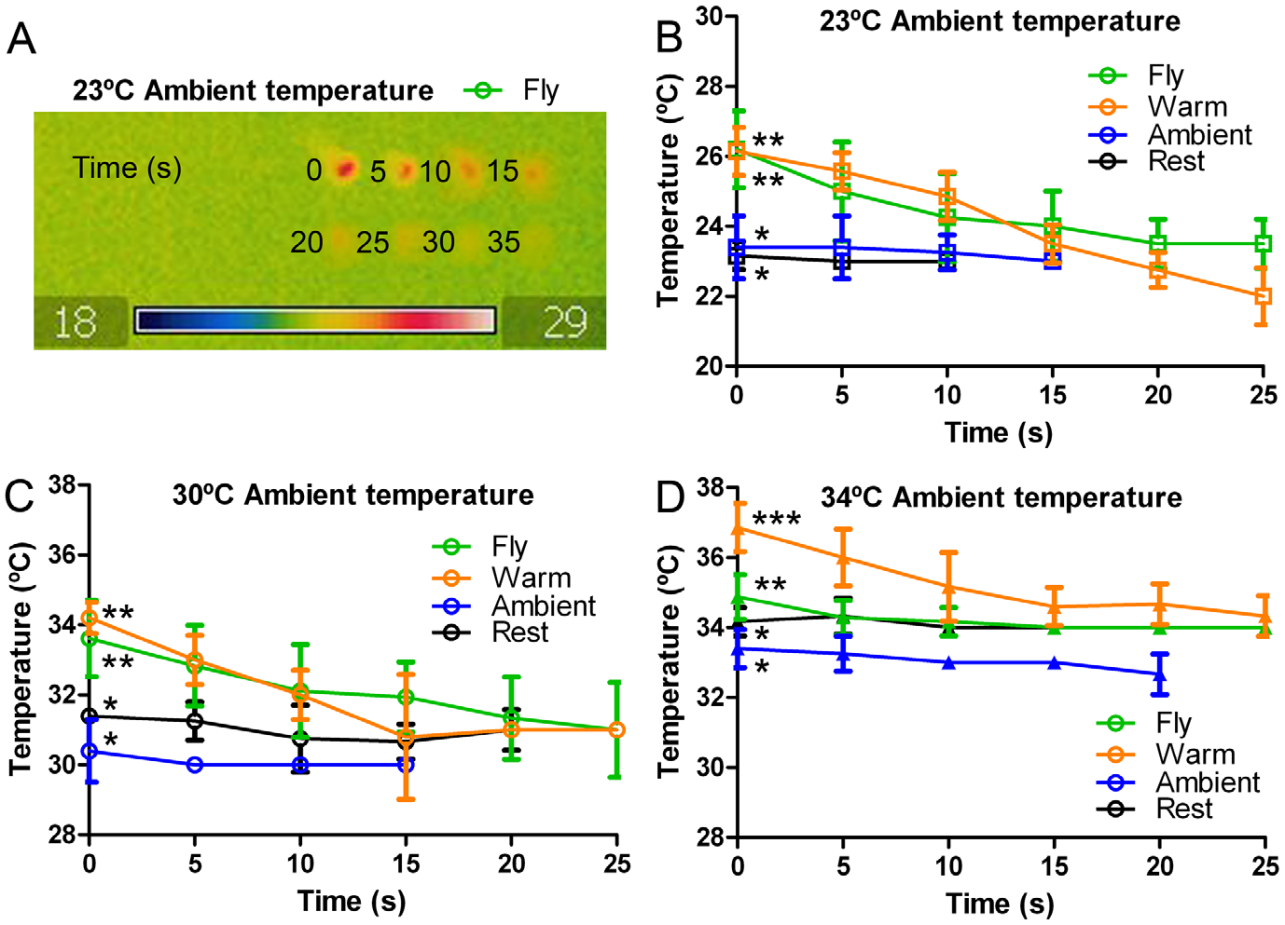
Thermal imaging data showing how the temperature of a *Trigona carbonaria* bee varies with time. (A) Composite image of a bee immediately after completing a flight (T = 0) and at 5 s intervals whilst the temperature of the bee cools until close to the ambient temperature. (B) Plots of Trigona mean (+/− s.d.) bee body temperature versus time starting immediately after completing one of four different activities [not flying for at least 5 min (resting), flying, drinking from a feeder that is warmer than the ambient temperature, and drinking from an ambient temperature feeder] at ambient temperatures of (B) 23°C, (C) 30°C and (D) 34°C. At an ambient temperature of 30°C the data for bee relative temperature is consistent with that observed at 23°C: at both 23°C (B) and 30°C (C) ambient temperatures a flying bee attains a temperature that is hotter than that of a resting bee, and drinking from a warm feeder allows bees to maintain these higher temperatures. At an ambient temperature of 34°C (D), drinking from a warm feeder raises bee temperature to well above what is maintained during active flight. This suggests that drinking warm nectar may be an advantage at lower ambient temperatures when it allows bees to maintain a flight temperature during the time it takes them to imbibe nectar, but at higher ambient temperatures the warmer nectar may cause the body temperature to exceed the flight range and no longer provide a benefit. For B–D at T = 0, plots with the same number of stars within a figure are not significantly different (P>0.05); plots with a different number of stars are significantly different (P<0.01; Mann Whitney U test).

There have been previous suggestions that warm flowers may offer insects an energetic benefit [4], [11], and this may explain the preference for the warmer feeder at ambient temperatures up to 30°C ambient temperature. The aim of Experiment 4 was to gain some empirical evidence for this hypothesis by examining the bees' body temperatures as well as to investigate what may underlie the change in their preferences at 34°C ambient temperature. Thermal imaging of bees after participating in one of four activities reveals that there are important differences depending upon the ambient temperature (Fig. 3). Bee temperatures were compared following resting, flying, imbibing ambient temperature sucrose or imbibing sucrose that is above ambient temperature. The results indicate that bees attain a body temperature several degrees higher than ambient when flying at the two lower ambient temperatures (23°C and 30°C; see Fig. 3, ‘Fly’ column), as found in previous studies [33]. However, this cools until it is close to ambient by 15–20 s after flight ceases (Fig. 4A–C). Consistent with this, resting bees have a body temperature the same as ambient (Fig. 3, ‘Rest’ column and Fig. 4 blue and black lines).

The body temperature of bees that have imbibed ambient temperature sucrose is not significantly different from that of bees resting at ambient temperature (Fig. 3, ‘Drink (amb)’ column). The temperature of resting bees is consistent with a flying bee that has landed and then rested for a period of at least 20 s (Fig. 4A). Importantly, at ambient temperatures of 23°C or 30°C, imbibing warm sucrose (Fig. 3, ‘Drink (warm)’ column; Fig. 4B and C, orange lines) generates a body temperature the same as that attained during flight (Fig. 3, ‘Fly’ column; Fig. 4B and C, green lines) and significantly higher than that of resting bees (Fig. 3, ‘Rest’ column; Fig. 4B and C, black lines).

The results change at an ambient temperature of 34°C (Fig. 3, bottom row and Fig. 4D). Again, there was no significant difference in temperature between resting bees and those imbibing ambient temperature nectar and both the flying bees and bees drinking from a warm feeder were at a temperature significantly above the ambient temperature (33.8°C+/−0.6). However, under these conditions, there was also a significant difference in temperature between flying bees (34.9°C+/−0.6) and bees drinking from a warm feeder (36.9°C+/−0.7).

To investigate the biological relevance of maintaining the flight body temperature by imbibing warm sucrose, the period of time bees rested when imbibing fluid was measured. As it takes 15–20 s from the cessation of flight for the body temperature to cool to ambient (Fig. 4), the fact that imbibing warm sucrose maintains the body temperature would not be biologically relevant if bees spent <15 s imbibing fluid. At an ambient temperature of 28°C the mean time was 20.7 s (+/− 3.7sd) and at an ambient temperature of 34°C the mean time bees imbibed solution was similar 18.9 s (+/− 3.1sd); an independent samples (t-test; t = 1.6, df38, p = 0.112) revealed imbibe time was not significantly different in the different ambient temperature conditions. This indicates that imbibing solution that is warmer than ambient assists in maintaining body temperature closer to that required for flight, so long as the ambient temperature does not exceed flight temperature.

## Discussion

### Warmth preference is shared by diverse bees

Bumblebees are able to perceive warmth as an additional reward to nutrition, and use cues like position or colour to identify flowers that are perceived as more rewarding [15], [16]. This study reveals that *T. carbonaria* also prefer warmer nectar at ambient temperatures up to 30°C and can use a spatial cue (position of the feeder) to find it. The results are consistent with the finding that bumblebees process sucrose sweetness and temperature independently [16], since the *T. carbonaria* preferences for a warmer feeder change in a dynamic way depending upon ambient temperature (Fig. 1). Given that stingless and bumblebees differ significantly in morphology and natural climate [18], [19] and shared their last common ancestor at least 65 million years ago [34], data from these two species suggests that a preference for warm flowers may be widespread amongst Apinae.

### Nectar temperature preference as a mechanism for bee thermal regulation

The optimal range for energetically efficient insect flight is relatively narrow, with a wider suboptimal range [35], [36]. The flight force produced by tethered honeybees peaks at a thoracic temperature of 38°C [37] and bumblebees regulate their thoracic temperature between 36 and 41°C over a wide range of ambient temperatures [36]. To continue foraging in varying conditions, insects have evolved mechanisms for moderating thoracic temperature [38]–[40]. This study establishes that the switch in sucrose temperature preference observed here is not due to aversion to a detrimentally hot stimulus. Furthermore, the results suggest that, at lower ambient temperatures, warm nectar (which is preferred) preserves the body temperature maintained during flight rather than allowing the cooling that would normally occur while the bee is in a relatively inactive state on a flower (Fig. 4). This is consistent with the suggestion that warmer nectar could offer an energetic benefit to pollinators [4], [5], [10]. In contrast, at 34°C ambient temperature, imbibing warmer nectar raises the body temperature above the normal flight temperature range (Fig. 4D) and ambient temperature nectar becomes the preferred choice (Fig. 1). Thus, this study indicates that flexibility in flower preference may be a novel behavioural mechanism for pollinator temperature homeostasis. Recent studies in *Drosophila* have shed light on the molecular and cellular mechanisms underlying insect temperature preference behaviour. Anterior cell neurons in the *Drosophila* brain are activated by the ion channel dTrpA1, which functions as a warmth sensor and has similar properties in other insects [41]. dTrpA1 is also expressed in the proboscis where it contributes to cold avoidance, providing a candidate for the mechanism of nectar temperature sensing in bees. cAMP-PKA signalling in the *Drosophila* mushroom bodies is also required for controlling temperature preference behaviour [42]. The orthologues of these genes may be important in controlling temperature preference in bees, and thereby regulate their flower preference behaviour to maintain an optimal thoracic temperature for flight.

The preferred sucrose temperature for *T. carbonaria* converges on approximately 34°C even with the option of a warmer feeder (Fig. 1). This temperature is in the range that the African stingless bees *T. denoiti* and *T. gribodi* actively maintain their respective hive temperatures (32–35°C) using air exchange ventilation, despite highly variable external temperatures [24]. This suggests it may be the ideal ambient temperature range for the *Trigona* genus. A similar range is likely to be physiologically important for free flying bees since honeybees start actively cooling their body via evaporation from their mouthparts if the ambient temperature exceeds approximately 34°C [39], [43]. The behavioural switch observed in *T. carbonaria* between ambient temperatures of 30–34°C may therefore be relevant to other important pollinator species.

### How might ambient temperature interact with pollination success?

Ambient temperature does not define floral temperature in the same way for all flowers. Heliotropism (tracking the sun) and morphological features such as colour and shape can raise intrafloral temperature up to 8°C above ambient [3], [5], [6], [44], [45], and evaporative cooling or self-shading can reduce temperature below ambient in very warm conditions [46]. Thermogenic plants use biochemical heat-generating pathways and evaporative cooling to maintain intrafloral temperature somewhat independently of ambient temperature [47]. For example, the sacred lotus *Nelumbo nucifera* switches from heat production to evaporative cooling at an ambient temperature of 34°C to maintain its floral temperature at 30–36°C [48]. Interestingly, this matches the preferences of *T. carbonaria* (Fig. 1) and the point at which honeybees begin evaporative cooling [39], [43].

Microclimatic factors such as ambient temperature, solar irradiance and wind speed can interact with a flower's morphological features to determine the intrafloral temperature and this can in turn influence pollination rates and the composition of various pollinators visiting the flowers [5], [49]. Even in thermogenic plants, there is great variation among species in how the rate of heat production and heat loss interacts with ambient temperature [47]. Given the variation in floral temperature regulation among plant species, it is likely that the relationship between flower temperature and ambient temperature will vary with climatic conditions, but not in exactly the same way for all species. Given the observation here of a switch point in pollinator preferences, this variation may in turn influence which flowers are preferred by pollinators in particular climatic conditions, and affect the relative reproductive success of different plant species.
